# Integrative analysis reveals a four-gene signature for predicting survival and immunotherapy response in colon cancer patients using bulk and single-cell RNA-seq data

**DOI:** 10.3389/fonc.2023.1277084

**Published:** 2023-10-31

**Authors:** Ruoyang Chai, Yajie Zhao, Zhengjia Su, Wei Liang

**Affiliations:** ^1^ Department of General Practice, Ruijin Hospital, Shanghai Jiaotong University School of Medicine, Shanghai, China; ^2^ Department of Geriatrics, Ruijin Hospital, Shanghai Jiaotong University School of Medicine, Shanghai, China

**Keywords:** colon cancer, single-cell RNA transcriptome, spatial transcriptome, bulk RNA transcriptome, differentially expressed genes, prognostic prediction, drug sensitivity

## Abstract

**Background:**

Colon cancer (CC) ranks as one of the leading causes of cancer-related mortality globally. Single-cell transcriptome sequencing (scRNA-seq) offers precise gene expression data for distinct cell types. This study aimed to utilize scRNA-seq and bulk transcriptome sequencing (bulk RNA-seq) data from CC samples to develop a novel prognostic model.

**Methods:**

scRNA-seq data was downloaded from the GSE161277 database. R packages including “Seurat”, “Harmony”, and “singleR” were employed to categorize eight major cell types within normal and tumor tissues. By comparing tumor and normal samples, differentially expressed genes (DEGs) across these major cell types were identified. Gene Ontology (GO) enrichment analyses of DEGs for each cell type were conducted using “Metascape”. DEGs-based signature construction involved Cox regression and least absolute shrinkage operator (LASSO) analyses, performed on The Cancer Genome Atlas (TCGA) training cohort. Validation occurred in the GSE39582 and GSE33382 datasets. The expression pattern of prognostic genes was verified using spatial transcriptome sequencing (ST-seq) data. Ultimately, an established prognostic nomogram based on the gene signature and age was established and calibrated. Sensitivity to chemotherapeutic drugs was predicted with the “oncoPredict” R package.

**Results:**

Using scRNA-Seq data, we examined 33,213 cells, categorizing them into eight cell types within normal and tumor samples. GO enrichment analysis revealed various cancer-related pathways across DEGs in these cell types. Among the 55 DEGs identified via univariate Cox regression, four independent prognostic genes emerged: PTPN6, CXCL13, SPINK4, and NPDC1. Expression validation through ST-seq confirmed PTPN6 and CXCL13 predominance in immune cells, while SPINK4 and NPDC1 were relatively epithelial cell-specific. Creating a four-gene prognostic signature, Kaplan-Meier survival analyses emphasized higher risk scores correlating with unfavorable prognoses, confirmed across training and validation cohorts. The risk score emerged as an independent prognostic factor, supported by a reliable nomogram. Intriguingly, drug sensitivity analysis unveiled contrasting anti-cancer drug responses in the two risk groups, suggesting significant clinical implications.

**Conclusion:**

We developed a novel prognostic four-gene risk model, and these genes may act as potential therapeutic targets for CC.

## Introduction

Based on global cancer statistics, colon cancer (CC) ranks as the third most prevalent malignancy and the fourth leading contributor to cancer-related fatalities worldwide (1). Predictions indicate 2.2 million new CC cases and 1.1 million CC-related deaths by 2030 (2). While innovative surgeries and targeted treatments have contributed to a reduction in CC mortality in recent years (3, 4), the disease’s subtle onset and aggressive progression often result in late-stage diagnoses, potentially leading to missed opportunities for optimal treatment (5–7).

Given the significant clinical variability inherent to CC, conventional clinical attributes such as the existing American Joint Committee on Cancer (AJCC) staging, Tumor-Nodal Involvement-Metastasis (TNM) staging, and tumor grades prove inadequate in precisely forecasting individualized prognoses (8–11). As a result, it becomes imperative to stratify CC patients and innovate novel markers to reliably predict both prognosis and therapy response. Over the last few decades, high-throughput sequencing technologies, such as bulk transcriptome sequencing (bulkRNA-seq), have emerged as powerful tools for identifying novel molecular biomarkers and advancing our comprehension of tumor development (12). Leveraging bulkRNA-seq data, significant efforts have been directed towards elucidating the utility of distinct gene signatures, including ferroptosis-related (13), RNA binding proteins (RBPs)-related (14), and immune-related gene signatures (15), for predicting CC prognosis. Notably, this encompasses our recent work on an ECM-related signature (16). However, tissue-level bulkRNA-seq predominantly focuses on the “average” expression across all cells, a limitation that hampers its capacity to capture the intricate molecular diversity within a tumor sample.

In contrast, single-cell transcriptome sequencing (scRNA-seq) is an innovative technology that reveals individual gene expression within cells, aiding in identifying cell subtypes and understanding variability (17). Recent advances in scRNA-seq help categorize colorectal cancer cells, explore gene differences, and distinguish between primary tumors and metastases (18–20). A few studies have effectively combined scRNA-seq and bulkRNA-seq data to establish and validate prognostic signatures in CC (21–26), such as identifying genes related to membrane tension and aging-related or autophagy-related genes (21–23).

Nevertheless, research on constructing a prognostic signature using DEGs across all cell types in the comparison between cancerous and normal samples is still limited.

In this study, our goal was to create a scRNAseq cell type-level DEGs-based prognostic model for CC patients. We identified DEGs across major cell types in tumor and normal samples, leading to a concise four-gene signature for predicting prognosis in COAD. We then validated the model in two independent cohorts, confirmed gene expression in scRNA-seq and single-cell spatial transcriptome sequencing (ST-seq) datasets [], and developed a practical nomogram incorporating the signature and clinical factors for 3-, 5-, and 10-year survival prediction. Additionally, we explored drug response differences in risk groups. Our findings offer a potential prognostic tool and therapeutic insights for CC prognosis.

## Methods

### Data sources

The single-cell transcriptome sequencing dataset (scRNA-seq) was downloaded from GSE161277 (18). The spatial transcriptome sequencing (ST-seq) dataset was downloaded from a spatial transcriptomics research website (http://www.cancerdiversity.asia/scCRLM/). The ST-seq data of two patients were used (27). The bulk RNA-sequencing (bulkRNA-seq) dataset was derived from the COAD cohort of The Cancer Genome Atlas (TCGA) and downloaded from GSE39582 and GSE33382.

### Single-cell RNA-seq analysis

**Figure 1:**
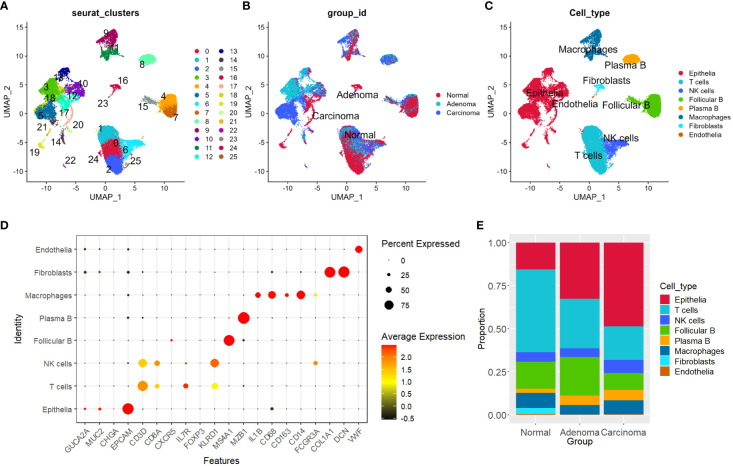
Distinguishing eight primary cell types in tumor and normal tissues. **(A–C)** UMAP plots depicting sample distributions, organized by Seurat clusters, group IDs, and cell types, respectively. **(D)** Dot plot illustrating expression levels and percentages of marker genes for each of the primary cell types. **(E)** Shift in proportions of each cell type observed across the three groups. UMAP, Uniform Manifold Approximation and Projection.

We utilized the “Seurat” R package (version 4.1.1) and applied standard downstream processing for scRNA-seq data (https://github.com/satijalab/seurat) (28). Genes that were detected in less than 3 cells as well as cells with less than 300 or more than 6,000 detected gene numbers were ruled out, and the mitochondria proportion was limited to less than 15%. Then, the LogNormalize method was applied for data normalization. Principal component analysis (PCA) was performed. The “Harmony” R package was then used to integrate all samples (29). Uniform Manifold Approximation and Projection (UMAP), a nonlinear dimensionality reduction method, was used for unsupervised clustering and unbiasedly visualizing cell populations on a two-dimensional map (30). Subsequently, for cell type annotation, we initially employed the “singleR” package (31) and subsequently verified the expression of established markers specific to various cell types (**Figures S1C, D**, and **Figure 1D**). Specifically, clusters 3, 5, 10, 12, 14, 17, 18, 19, 20, and 21 were characterized as epithelial cells by the expression of epithelial marker EPCAM; clusters 0, 1, 2, 24, and 25 were identified as T cells expressing CD3D, CD8A and IL7R; clusters 6 was identified as natural killer cells (NK cells) expressing KLRD1; clusters 4, 7, and 15 were follicular B cells due to the expression of MS4A1; cluster 8 was plasma B cells expressing MZB1; clusters 9 and 11 were macrophages corresponded to expression of CD68, CD14 and FCGR3A; cluster 16 was identified as fibroblasts expressing COL1A1 and DCN; and cluster 23 was marked as endothelial cells by expression of VWF (**Figure S1D**). The “FindlMarkers” function was utilized to identify differentially expressed genes (DEGs) of each cell type. In addition, the expression pattern of genes was visualized by applying the “FeaturePlot” function in “Seurat” and functions in “ggplot2”.

### Spatial transcriptomics data processing

For the ST-seq data analysis, we utilized the “Seurat” R package (v4.1.1). The data underwent log-normalization for standardization (28). “RunPCA” was employed to run PCA. Subsequently, the FindNeighbors and FindClusters functions were applied to identify clusters of similar ST spots. Preliminary annotations of distinct clusters were performed based on hematoxylin-eosin (H&E) staining sections and unsupervised clustering analysis. To enhance accuracy, the final annotation of clusters was aligned with findings from previous studies (18). In addition, the expression pattern of identified genes was visualized by applying the “SpatialFeaturePlot” function in “Seurat”.

### Gene ontology analyses

To gain comprehensive insights into the distinctions between various cell types within cancerous and normal samples, we conducted GO analysis on the set of DEGs specific to each cell type. These DEGs were subjected to enrichment analysis using the Metascape platform, facilitating the exploration of gene ontology term enrichment within each cell type (https://metascape.org/gp/index.html#/main/step1) (32). This encompassed the assessment of Biological Processes (BP), Cellular Components (CC), and Molecular Functions (MF). We then used the “ggplot2” R package to visualize the results.

### Construction and verification of the prediction model

We utilized normalized RNA-seq data and clinical information from The Cancer Genome Atlas (TCGA) training cohort, which included overall survival time and status, for constructing and verifying our prediction model. Initially, we conducted univariate Cox regression analysis to identify candidate DEGs with significant prognostic value (P < 0.05). To prevent model overfitting, we employed the “glmnet” package to perform least absolute shrinkage operator (LASSO) analyses regression analysis, further refining the selection of prognostic genes (TRAM1, DNM2, TRAF5, NPDC1, PTPN6, VEGFA, TBX2, SPINK4, PSMB2, RPS24, CXCL14, CNKSR3, CXCL13, KRTCAP3, SPINK1, RHOBTB3, CA4, TPM4, PCCA, EIF3F, ZFAND2A, UGT2B17, HSPA1B, CD177, CEBPD, and CD24) (33, 34). Cox proportional hazard regression was then utilized to finalize the optimal prognostic genes for the model. The formula for the gene signature was formulated as follows: risk score = Σ (βi * Exp.i) (where i denotes the number of prognostic genes, βi represents the coefficient of gene i, and Exp.i represents the expression level of gene i). Subsequently, we categorized colon cancer (CC) patients into high- and low-risk groups based on the median risk score within each dataset.

To assess the predictive performance of the established prognostic signature, Kaplan-Meier survival curve analysis was employed. This analysis aimed to determine whether a significant disparity existed in overall survival time between the high- and low-risk groups. The “survival” and “survminer” R packages were utilized for this purpose, and the log-rank test (P < 0.05) was applied to ascertain statistical significance. Additionally, the predictive capability of the prognostic signature was evaluated through time-dependent receiver-operating characteristic (ROC) curve analysis, facilitated by the “timeROC” and “survival” R packages. Furthermore, we validated the prediction model’s performance using the independent validation cohort.

### Development and validation of nomogram

Following the multivariate Cox proportional hazard regression analysis, we employed the “rms” R package to construct a nomogram. This nomogram was designed to predict the survival outcomes of patients with colon adenocarcinoma (COAD) at 3-, 5-, and 10-year intervals. The nomogram was created by integrating the risk model associated with the four-gene signature and the clinical variable of age. Subsequently, we utilized calibration curves and time-dependent receiver-operating characteristic (ROC) analysis to assess the predictive accuracy and performance of the developed nomogram.

### Drug sensitivity analysis

To identify potential drug susceptibility patterns in the GDSC2 database and predict drug responses in the context of COAD, we employed the “oncoPredict” R package (35). Visualization of the scatterplot was achieved using the “ggplot2” package, facilitating a comprehensive understanding of the relationship between drug response and risk outcomes.

### Statistical analysis

R software (R: version 4.1.2.; RStudio: 2022.02.3 Build 492) was employed for data processing (https://www.r-project.org/). The “FindlMarkers” function was utilized to identify DEGs of each cell type with the filter value of absolute log2 fold change (FC) ≥ 0.5 and the minimum cell population fraction in both of the populations was 0.1. “MAST” was used for statistical analysis. Kaplan-Meier analysis was performed to assess survival differences between high- and low-risk groups, and the log-rank test (P < 0.05) was applied to ascertain statistical significance. In drug sensitivity analysis, we employed the Wilcox test as the statistical measure, considering P < 0.05 as significant.

## Results

### scRNA-seq data analysis and cell type annotation

The figures in **Supplementary Figures 1A, B** present the distribution of total gene numbers, total count numbers, and percentages of mitochondria genes for single cells from each individual sample. The “Seurat” pipeline (28) identified 26 distinct clusters across the normal, adenoma, and carcinoma samples from the four patients (**Figures 1A–C**, Methods). We utilized a combination of automated and manual annotation methods to categorize the 26 clusters into eight major cell types (see Methods), comprising epithelial cells, T cells, natural killer cells (NK cells), follicular B cells, plasma B cells, macrophages, fibroblasts, and endothelial cells (**Figures 1C, D**, and **S1C, D**) Subsequently, we quantified the composition of each cell type within different sample groups. Our observations revealed a noticeable increase in the proportion of epithelial cells in both adenoma and carcinoma samples, whereas the presence of T cells exhibited a decrease (**Figure 1E**).

### Enrichment analysis of DEGs for major cell types

**Figure 2:**
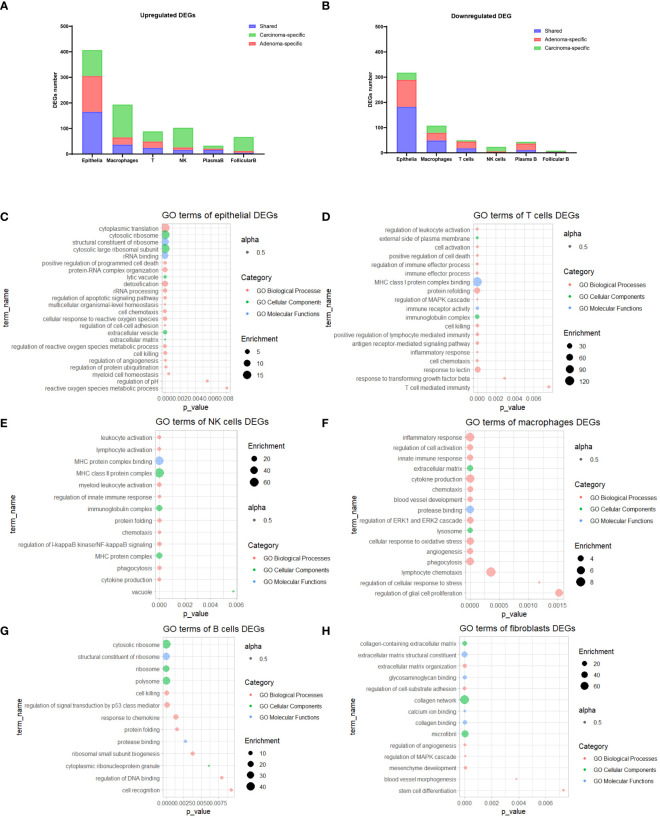
GO Enrichment analysis of DEGs. **(A, B)** Stacked bar plots illustrating the count of DEGs for each cell type. **(C-H)** Bubble plots showcasing the most prominent enriched GO terms for DEGs within epithelial cells **(C)**, T cells **(D)**, NK cells **(E)**, macrophages **(F)**, B cells **(G)**, and fibroblasts **(H)**. GO, Gene Ontology; DEGs, Differentially Expressed Genes.

Next, to gain deeper insights into the differences in each cell type between cancerous and normal samples, we initially identified DEGs (**Figures 2A, B**) and then conducted GO analysis on the DEGs specific to each cell type. Notably, in the case of epithelial DEGs, enrichment was observed in the realm of cytoplasmic transition, protein-RNA complex organization, extracellular matrix, and angiogenesis (**Figure 2C**). For both T cells and NK cells, gene enrichment predominantly revolved around cell activation processes, cell chemotaxis, and MHC protein complex binding (**Figures 2D, E**). Furthermore, the DEGs identified in macrophages exhibited enrichment in terms of inflammatory responses and genes linked to phagocytosis activity (**Figure 2F**). B cells displayed enrichment of genes associated with ribosomal activity and cell killing (**Figure 2G**), whereas fibroblasts demonstrated enrichment in genes related to collagen-related extracellular matrix organization and function (**Figure 2H**). Corresponding networks of major GO terms for each cell type are shown in **Figure S2** (**Figures S2A–F**).

### Prognostic genes identification

**Figure 3:**
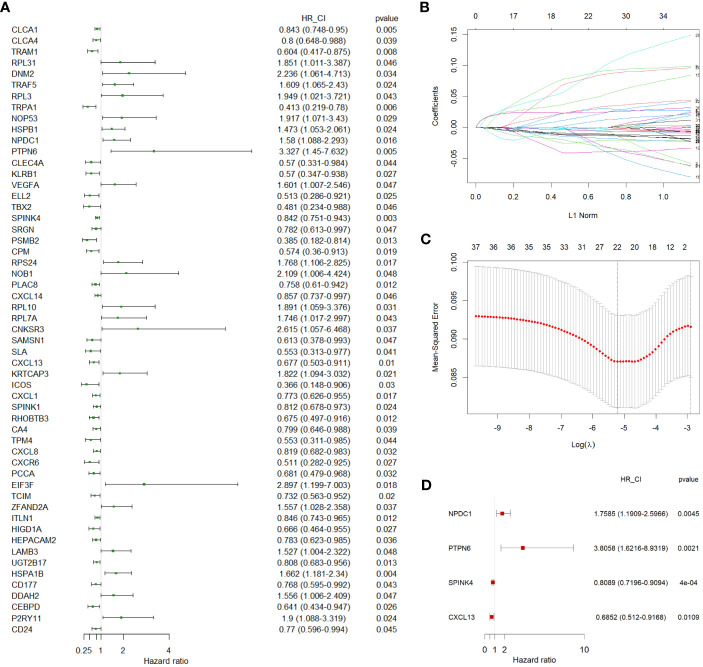
Identification of independent prognostic genes. **(A)** A forest plot showing 55 prognostic genes identified by univariate Cox regression. **(B, C)** LASSO regression analysis on 55 prognostic genes. **(D)** A forest plot showing the 4 independent prognostic genes identified by multivariate Cox regression. LASSO, least absolute shrinkage operator.

In our pursuit of a comprehensive assessment of the prognostic implications of all DEGs across distinct cell types identified in the scRNA-seq data for CC, we leveraged TCGA-COAD as our training cohort. Initially, we executed univariate Cox regression analysis to explore their prognostic relevance. This analysis revealed a subset of 55 DEGs that displayed a statistically significant correlation with the prognosis of CC patients, evidenced by P values < 0.05 (**Figure 3A**). We next employed LASSO regression to circumvent potential model overfitting (See Methods, **Figures 3B, C**). Subsequently, we undertook multivariate Cox regression analysis on the identified 24 candidate genes, aiming to ascertain their roles as independent prognostic factors. Ultimately, our analysis revealed four genes - NPDC1, PTPN6, SPINK4, and CXCL13 - as potentially constituting a prognostic signature (**Figure 3D**). The subsequent step involved the calculation of a risk score for each CC patient, which was derived from the following formula: Risk Score = 0.56448 * Expression of NPDC1 + 1.33652 * Expression of PTPN6+ (-0.21203) * Expression of SPINK4+ (-0.37809) * Expression of CXCL13.

### Expression pattern of identified genes in scRNA-seq dataset and ST-seq dataset

**Figure 4:**
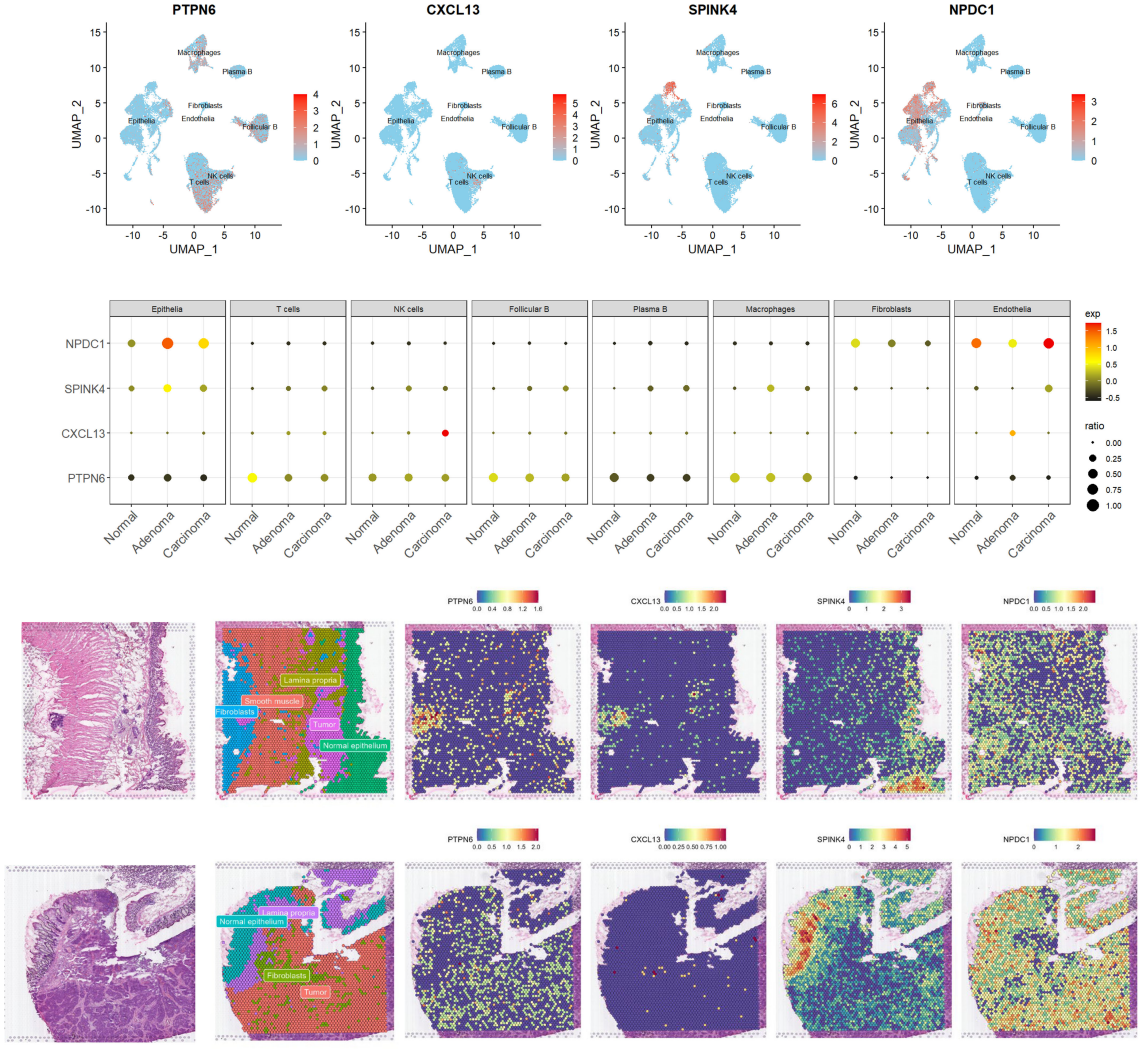
Expression profiling of four genes in scrna-seq and ST-seq datasets. **(A, B)** Feature plots **(A)** and dot plot **(B)** offering a visual representation of the expression profiles of the four identified genes within the scRNA-seq dataset. **(C, D)** Spatial feature plots showing the distinct expression patterns of the same four genes within two ST-seq datasets. scRNA-seq, single-cell transcriptome sequencing; ST-seq, spatial transcriptome sequencing.

To gain a more comprehensive insight into the expression patterns of the four prognostic genes we identified, we meticulously checked their expression patterns in the scRNA-seq data first. Our analysis unveiled that the expression of PTPN6 was a pervasive presence across the various cell types. Notably, it exhibited a distinctive prominence within immune cell populations, including T cells, NK cells, macrophages, and B cells (**Figure 4A**). Intriguingly, its expression was downregulated in these cell types within tumor samples (**Figure 4B**). Fascinatingly, CXCL13’s primary expression was observed in NK cells (**Figure 4A**), and this expression was elevated in NK cells within tumor tissues, particularly in carcinomas (**Figure 4B**). Furthermore, both NPDC1 and SPINK4 were significantly enriched in epithelial cells, although NPDC1 demonstrated a more extensive expression profile (**Figure 4A**). Remarkably, both genes exhibited increased expression in epithelial cells within tumor tissues (**Figure 4B**).

Expanding our investigation, we also harnessed the power of ST-seq. Initially, we performed cell type annotation in the ST-seq data using a combination of previously published research and the expression patterns of marker genes (**Figures 4C, D**, and **S3A, B**). Remarkably, there was a noteworthy degree of overlap in the expression profiles of PTPN6 and CXCL13, particularly evident in patient 1 (**Figures 4C, D**). However, PTPN6 exhibited a broader and more widespread distribution across the studied context. Our findings aligned with the scRNA-seq data, revealing that those dots exhibiting high CXCL13 expression also co-expressed T cell markers such as CD3D and IL7R (**Figures S3A, B**). Interestingly, SPINK4’s expression landscape predominantly corresponded to a cell type annotated as normal epithelia (**Figures 4C, D**). Conversely, NPDC1 demonstrated a more universal presence across various tissue components (**Figures 4C, D**). To a certain extent, the expression patterns of these four genes were consistently observed across both datasets. This intricate exploration encompassing diverse datasets collectively enhances our comprehension of the expression patterns and plausible functional roles of these prognostic genes.

### Verification of accuracy of four-gene signature in CC

**Figure 5:**
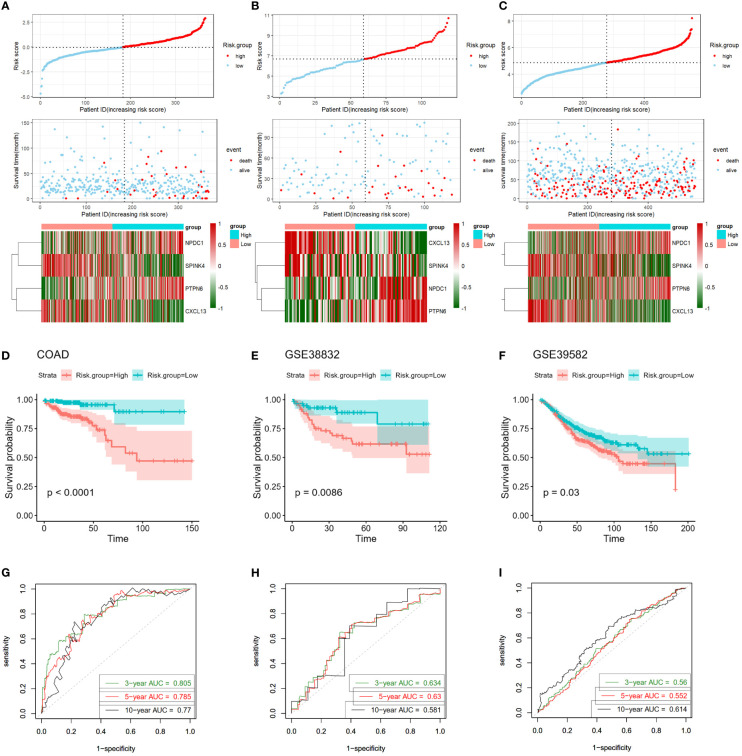
Validation of four-gene signature in training and validation cohorts. **(A–C)** Upper Panels: Visualization of risk score distribution derived from the four-gene prognostic signature in both COAD and validation cohorts. Middle Panels: Illustration of survival status of colon cancer patients categorized by high- or low-risk scores in both COAD and validation cohorts. Lower Panels: Heatmaps displaying expression patterns of the four genes constituting the prognostic signature in COAD and validation cohorts. **(D–F)** Kaplan-Meier survival curves exhibit markedly shorter survival times in high-risk groups compared to low-risk score groups, evident in both COAD and validation cohorts. **(G–I)** Time-dependent ROC curves assess the prognostic performance of the four-gene signature in COAD and validation cohorts. COAD, colon adenocarcinoma; ROC, receiver-operating characteristic.

Subsequently, we validated the accuracy and efficacy of our four-gene signature model across multiple cohorts, encompassing the training cohort (COAD) and two independent validation cohorts (GSE33882 and GSE39582). For each CC patient, an individual risk score was calculated using the established four-gene signature model. By leveraging these risk scores, we segregated all CC patients into high- and low-risk groups, dichotomized at the median risk score value within each cohort (also shown in upper panels in **Figures 5A–C**). A compelling visual representation emerged as we ranked patients from low to high based on their risk scores. Scatter plots indicated a distinct survival pattern, with low-risk patients exhibiting considerably improved survival rates compared to their high-risk counterparts (as illustrated in the middle panels in **Figures 5A–C**). Notably, red dots within the scatter plots symbolized patients who were deceased, while blue dots represented those who remained alive. Furthermore, heat maps were deployed to illustrate the differential expression profiles of the four signature genes between the high- and low-risk groups (as shown in the lower panels in **Figures 5A–C**).

To deepen our understanding, we engaged in Kaplan-Meier survival curve analyses. The outcomes strikingly underscored that those patients allocated to the high-risk group experienced significantly poorer overall survival outcomes compared to those in the low-risk group, a trend consistent across both the training cohort (log-rank P < 0.001; **Figure 5D**) and validation cohorts (GSE33882: P = 0.00086; GSE39582: P = 0.03, **Figures 5E, F**).

Intriguingly, the predictive prowess of our signature model was reinforced by the AUC calculated from ROC curves. For the prediction of 3-, 5-, and 10-year overall survival in the training cohort, the AUCs were 0.805, 0.785, and 0.777, respectively (**Figure 5G**). Correspondingly, the GSE33882 cohort exhibited AUCs of 0.634, 0.63, and 0.581 for the 3-, 5-, and 10-year overall survival predictions (**Figure 5H**). The GSE39582 cohort presented AUCs of 0.56, 0.552, and 0.614 for the 3-, 5-, and 10-year overall survival predictions, respectively (**Figure 5I**). Collectively, these compelling findings underline the robustness of our four-gene signature in distinguishing the prognostic trajectories of CC patients across multiple cohorts.

### Association between the risk score and clinical features of CC patients

**Figure 6:**
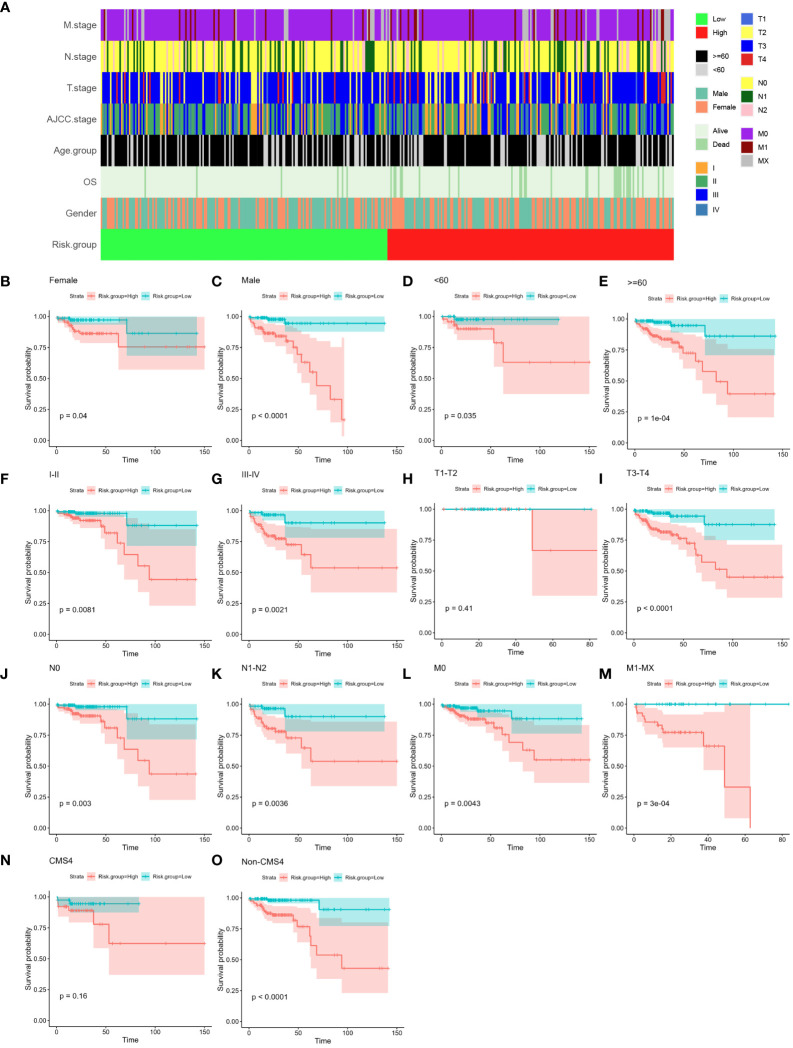
Relationship between established four-gene signature and clinical features. **(A)** Tile plot visually displaying the correlation between risk scores and various clinical features among CC patients in the COAD cohort. These features include age, gender, AJCC stage, T stage, N stage, and M stage. **(B–O)** Subgroup Kaplan-Meier curve analysis presenting the overall survival probabilities of high- and low-risk CC patients in the COAD cohort. The analysis is stratified based on different factors: age (<60, ≥60), gender (female, male), AJCC stage (stage I/II, stage III/IV), T stage (T1-T2, T3-T4), N stage (N0, N1-T2), M stage (M0, M1-MX) and CMS subtype (CMS4 and Non-CMS4). The log-rank test was used to calculate statistical significance. CC, colon cancer; COAD, colon adenocarcinoma; AJCC, American Joint Committee on Cancer; T stage, Tumor stage; N stage, Nodal Involvement stage; M stage, Metastasis stage; CMS subtype, consensus molecular subtype.

Examining the relationship between the four-gene risk score and various clinical attributes of colon cancer (CC) patients constituted our subsequent exploration. Firstly, the relationships of all clinicopathological classifications of all samples were depicted in **Figure 6A**, unraveling a multi-layered perspective. Subsequently, leveraging the risk score, as well as patient age, gender, AJCC stage, T stage, N stage, and M stage, facilitated the stratification of individuals into high- and low-risk groups (**Figures 6B–O**). The ensuing Kaplan-Meier analyses vividly portrayed significant prognostic disparities across these groups, underscoring their clinical relevance. Particularly, notable disparities were evident across the majority of clinical features. However, it is important to highlight that for the T1-T2 stage and CMS4, while the distinction did not reach statistical significance, a discernible trend was still observable. This trend could potentially be attributed to the limited sample size in these categories (P > 0.05; as illustrated in **Figures 6H–N**). Together, these findings collectively reinforce the predictive potential of the four-gene-based risk score model, which exhibited promising prognostic utility across various clinical features in CC patients.

### Four-gene prognostic signature is an independent prognostic factor

**Figure 7:**
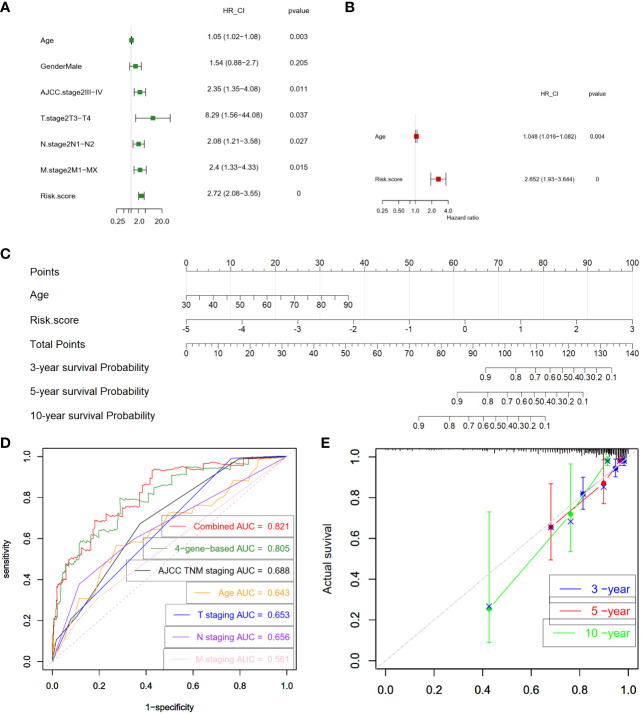
Construction and validation of prognostic nomogram. **(A)** Analysis of individual feature contribution through Univariate Cox regression in the training cohort. **(B)** Examination of gene significance after adjusting for other factors in the training cohort using Multivariate Cox regression. **(C)** Development of a prognostic nomogram utilizing the risk score from the four-gene signature and clinical factors. This nomogram predicts the overall survival rates of COAD patients at 3-, 5-, and 10-year intervals. **(D)** Assessment of the prognostic capability of the nomogram through time-dependent ROC curves in the COAD cohort. **(E)** Presentation of time-dependent calibration curves illustrating the alignment between predicted and observed 3-, 5-, and 10-year survival rates. COAD, colon adenocarcinoma; ROC, receiver-operating characteristic; HR, hazard ratio; 95% CI, 95% confidence interval; AJCC, American Joint Committee on Cancer; T stage, Tumor stage; N stage, Nodal Involvement stage; M stage, Metastasis stage; AUC, area under the curve.

Next, we examined whether the four-gene prognostic signature serves as an independent predictor for the survival outcomes of CC patients. To this end, both univariate and multivariate Cox regression analyses were executed, encompassing pertinent clinical features - age, gender, AJCC stage, T stage, N stage, and M stage - alongside the risk scores of patients within the training cohort. The univariate analysis shed light on the influence of various factors on overall survival (OS). The findings indicated that age (P = 0.003), AJCC stage III-IV (P = 0.011), T3-T4 stage (P = 0.037), N1-N2 stage (P = 0.027), M1-MX stage (P = 0.015), and risk score (P < 0.0001) exhibited significant correlations with OS within the training set (**Figure 7A**). Subsequently, the multivariate analysis delved deeper into the interplay of these factors. Remarkably, age (P = 0.004) and the risk score (P < 0.0001) retained their significant correlations with overall survival in CC patients, even when considered in the presence of other clinical attributes (**Figure 7B**). Collectively, these findings substantiate the conclusion that the four-gene prognostic signature stands as a pivotal independent factor significantly influencing the prognosis of CC patients, emphasizing its clinical relevance and potential utility as a prognostic predictor.

### Construction and validation of a nomogram

Subsequently, we proceeded to formulate a nomogram that amalgamates multiple prognostic predictors including age and risk score to comprehensively evaluate the probabilities of 3-, 5-, and 10-year overall survival in CC patients within the training cohort (**Figure 7C**). Notably, our findings highlighted the pivotal influence of the risk score on the prediction of overall survival. In **Figure 7D**, our analysis of ROC curves revealed a compelling outcome. Specifically, the 3-year Area AUC value for the four-gene risk score model was an impressive 0.805, surpassing the predictive potential of individual clinical factors, including the AJCC TNM stage (AUC = 0.688), patient’s age (AUC = 0.643), T stage (AUC = 0.653), N stage (AUC = 0.656), and M stage (AUC = 0.561) (**Figure 7D**). Furthermore, when we conducted a comprehensive ROC analysis, integrating the risk score with age, the resultant ROC curve exhibited a notably enhanced performance (AUC = 0.821) compared to each parameter in isolation. Moreover, the calibration curve provided additional validation, showcasing a satisfactory agreement between the predictions and actual observations across the probabilities of 3-, 5- and 10-year OS (**Figure 7E**). Collectively, these compelling findings highlight the potential of the nomogram, enriched by the risk score, to accurately predict the 3-, 5- and 10-year overall survival rates of CC patients. This integrated approach offers valuable insights for tailoring individualized clinical treatment strategies for CC patients.

### Drug sensitivity prediction in CC patients in the high- and low-risk groups

**Figure 8:**
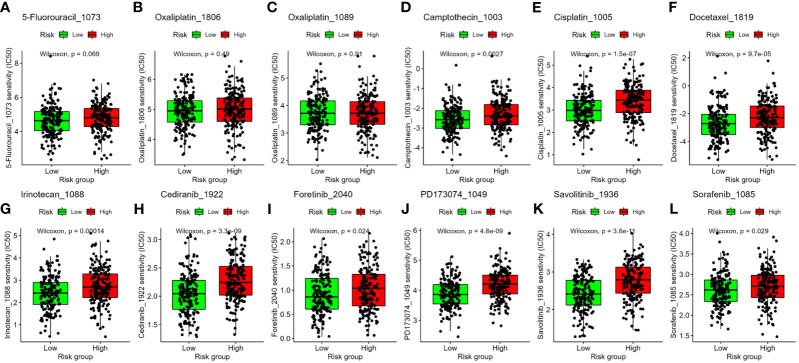
Association between the four-gene signature and drug sensitivity, including chemotherapeutics **(A–G)** and small molecular drugs targeting RTK pathway **(H–L)**. IC50: half-maximal inhibitory concentration. RTK, receptor tyrosine kinase.

To evaluate our four-gene signature’s clinical application and identify relevant drugs for high-risk patients, we analyzed chemotherapeutic sensitivity. The IC50 values for 5-fluorouracil and oxaliplatin showed no risk group difference (**Figures 8A–C**). However, Camptothecin_1003, Cisplatin_1005, Docetaxel_1819, and Irinotecan_1088 had significantly lower IC50 values in the low-risk group, implying heightened sensitivity (**Figures 8D–G**). Compounds such as Cediranib_1922, Foretinib_2040, PD173074_1049, Savolitinib_1936, and Sorafenib_1085, targeting the receptor tyrosine kinase (RTK) pathway, were projected to be more effective in the low-risk group as well (**Figures 8H–L**).

## Discussion

CC has emerged as a significant public health concern in recent decades, marked by increasing morbidity and mortality rates (1). To enhance personalized treatment strategies, the development of robust prognostic models is imperative. Extensive efforts have been dedicated to identifying CC biomarkers, often accomplished by scrutinizing differential gene expression between tumor and normal tissues through bulkRNA-seq analysis (13–16). Nonetheless, the cellular diversity intricacies of normal and tumor tissues were overlooked by bulkRNA-seq methods. scRNA-seq, an innovative methodology, allows us to precisely distinguish different cell types within tissues, providing a powerful tool to understand the complex mix of cell populations when comparing, for instance, tumor and normal tissues (18, 20). Additionally, scRNA-seq has the potential to uncover crucial hub genes related to tumor initiation and cancer advancement, which could be instrumental in shaping personalized therapies for CC patients (21–26, 36).

In this study, we conducted a comprehensive analysis of scRNA-seq data derived from 33,213 high-quality cells, sourced from the dataset GSE161277 (18). Our analysis highlighted the presence of significant heterogeneity within cells present in normal tissues, as well as tumor tissues. Employing the widely used “Seurat” pipeline, we successfully identified 26 distinct clusters. Leveraging a dual approach involving “singleR”-based auto-annotation and marker expression-guided manual annotation, we annotated these clusters into eight major cell types: epithelial cells, T cells, NK cells, macrophages, plasma B cells, follicular B cells, fibroblasts, and endothelial cells. Remarkably, our analysis unveiled notable alterations in the proportion of these cell types between cancerous and normal tissues. Specifically, we noted a significant increase in the proportion of epithelial cells within both adenoma and carcinoma samples, while the proportion of T cells decreased, consistent with a previous study (19). Subsequently, we investigated the gene alteration across the primary cell types in CC and normal tissues. Notably, the most pronounced changes were observed in epithelial cells, displaying the largest number of DEGs, followed by macrophages. Through GO enrichment analysis, we unveiled the distinct pathways enriched among these eight cell types. For instance, the DEGs in epithelial cells were predominantly associated with cytoplasmic translation processes. In T cells and NK cells, the DEGs pointed to heightened activity in leukocyte activation and immune responses. Macrophages showed enrichment in inflammatory response-related terms, while altered genes in fibroblasts were linked to pathways involving extracellular matrix organization, as expected. This comprehensive analysis provides insights into the functional shifts occurring in diverse cell populations during CC development.

Integrating COAD bulkRNA-seq data with clinicopathological data, we identified 55 prognostic genes from all DEGs, refining them to four (PTPN6, CXCL13, SPINK4, and NPDC1) through LASSO regression and multivariate analysis. The expression pattern of these four identified prognostic genes was then verified by scRNA-seq and ST-seq data, which revealed their distinct enrichments: PTPN6 and CXCL13 in immune cells, NPDC1 and SPINK4 in epithelial cells. We established and rigorously validated a four-gene prognostic signature using a risk score based on gene expression. Stratifying patients into high- and low-risk groups revealed significant survival differences, affirming our model’s clinical relevance.

Our investigation revealed a notable pattern: protein tyrosine phosphatase, non-receptor type 6 (PTPN6, also known as SHP-1), displayed prominent expression in immune cells, encompassing T cells, NK cells, and macrophages, aligning with prior findings (37). Intriguingly, its expression levels within these cell types were diminished in both adenoma and carcinoma samples in comparison to normal samples. T cells, specifically CD8+ T cells and CD4+ T cells, hold pivotal roles in adaptive immune responses. With a 95% homology shared between human and mouse PTPN6 (38), its absence in T cells has been linked to the establishment of more stable and enduring synapses with antigen-presenting cells in CD8+ T cells (38). This, in turn, yields lowered activation thresholds and heightened T cell proliferation. The exploration of strategies capitalizing on PTPN6 abrogation has yielded promising results. Noteworthy pre-clinical studies showcased the benefits of transferring PTPN6 knockout T cells in leukemia models (39). Meanwhile, two phase I clinical trials have evaluated the safety and potential of systemic treatment with sodium stibogluconate (SSG), a licensed leishmaniasis treatment that also acts as an active-site inhibitor of both PTPN6 and the related SHP-2 (40, 41). These trials aimed to harness this property as a plausible cancer therapy. In contrast, a significant increase of chemokine C-X-C motif ligand 13 (CXCL13) was observed solely in CD8+ NK cells in both adenoma and carcinoma samples in our analyses. This finding aligns with a recent study that integrated single-cell and spatial transcriptome analyses, unveiling an enrichment of CD8+/CXCL13+ T cells in CC and liver metastatic tumors (19). This suggests their potential role as a tumor-activating subset. Stratifying CC patients from the GEO cohort GSE39582 into CXCL13-high and CXCL13-low groups, the aforementioned study unveiled a favorable overall survival prediction in those with higher CXCL13 expression in CC (19), consistent with our findings in a previous study and this study (16). It is worth noting that our analysis of COAD bulkRNA-seq data in a previous study indicated downregulated CXCL13 in tumor samples (16). This discrepancy may be attributed to the intricate nature of tissue-level bulk-seq analyses, wherein scRNA-seq offers a more lucid understanding. However, rigorous experimental exploration is imperative to delve deeper into the role of CXCL13 in CC.

Contrasting with PTPN6 and CXCL13, our investigation revealed SPINK4 and NPDC1 to be predominantly enriched in epithelial cells rather than immune cells. Both genes exhibited upregulation in epithelial cells from cancerous tissues. Interestingly, a previous study has also found that the expression of serum SPINK4, the serine peptidase inhibitor Kazal type 4 gene, in patients with CC is elevated, and this increased expression has a high diagnostic value (42). Research by Wang et al. further showed that the downregulation of SPINK4 is associated with poor survival in CC patients and a high TNM stage, which is consistent with our data (43). In contrast, Chen and colleagues showed that high expression of SPINK4 is related to the advanced clinicopathological characteristics and poor treatment response of rectal cancer patients receiving chemotherapy (44). NPDC1, the neural proliferation, differentiation, and control 1 gene, encodes a 34-kDa protein predominantly expressed in neural tissues. Notably, in 1995, Galiana and colleagues observed that NPDC1 overexpression led to a notable inhibition of cell proliferation (45). Despite being relatively less explored, NPDC1 has garnered attention due to its significant upregulation during acute myeloid leukemia (AML) relapse. Intriguingly, a recent investigation has established a direct link between elevated NPDC1 expression and an adverse prognosis in AML cases (46).

Furthermore, we constructed a nomogram that integrated age and the risk score, enabling the prediction of 3, 5, and 10-year overall survival probabilities for CC patients. Notably, the predictive efficacy of this combined nomogram surpassed that of individual factors alone, such as age and AJCC TNM stage. By stratifying patients based on the final risk score, we observed significant disparities in drug sensitivity between low- and high-risk patients, particularly in response to certain chemotherapeutic agents and molecular-targeted drugs. These findings suggest that the risk signature holds the potential to guide the selection of chemotherapy and targeted therapy. Ultimately, leveraging this innovative four-gene signature, an approach combining immunotherapy, chemotherapy, and targeted therapy could be optimized for the tailored treatment of CC patients.

Notwithstanding the promising findings, this study does have certain limitations. Firstly, our examination of gene prognostic performance was confined to the RNA level; exploring protein-level implications demands further inquiry. Secondly, the use of a limited number of marker genes could introduce noise into practical applications; substituting them with more extensive sets of markers could enhance score reliability without substantial accuracy compromise. Thirdly, some more advanced techniques, such as machine learning, can be used to improve the accuracy of the prediction (47–49). Lastly, our analysis relied on bioinformatics approaches; additional cell or animal experiments are requisite to unveil the prospective roles of the identified genes in the progression of colon cancer.

## Conclusions

In conclusion, our integration of scRNA-seq data with validated cohorts highlights the robust prognostic and drug sensitivity predictive capabilities of the identified four DEGs in CC patients. This four-gene model holds promise as a valuable prognostic tool, aiding clinical decision-making by identifying patients who could potentially benefit from targeted anticancer drug therapies.

## Data availability statement

The original contributions presented in the study are included in the article/**Supplementary Material**. Further inquiries can be directed to the corresponding author.

## Ethics statement

Ethical approval was not required for the study involving humans in accordance with the local legislation and institutional requirements. Written informed consent to participate in this study was not required from the participants or the participants’ legal guardians/next of kin in accordance with the national legislation and the institutional requirements.

## Author contributions

WL: Conceptualization, Data curation, Funding acquisition, Investigation, Project administration, Resources, Supervision, Writing – original draft, Writing – review & editing. RC: Conceptualization, Data curation, Formal Analysis, Investigation, Methodology, Resources, Software, Validation, Visualization, Writing – original draft, Writing – review & editing. YZ: Data curation, Formal Analysis, Investigation, Methodology, Validation, Visualization, Writing – original draft, Writing – review & editing. ZS: Data curation, Formal Analysis, Investigation, Methodology, Validation, Visualization, Writing – original draft, Writing – review & editing.

## Supplementary material

Supplementary Figure 1 Automated annotation using “singleR”. **(A)** Overview of Gene Count and Mitochondrial Gene Proportion in All Samples. **(B)** Detailed information on each scRNA-seq sample downloaded in this study. **(C)** UAMP plot based on “singleR” annotation. **(D)** Dot plot illustrating expression levels and percentages of marker genes for each of the clusters. UMAP, Uniform Manifold Approximation and Projection; scRNA-seq, single-cell RNA sequencing.

Supplementary Figure 2 Enriched term networks by cluster ID. **(A-F)** Networks illustrating enriched terms for specific cell clusters: **(A)** Epithelial Cells, **(B)** T Cells, **(C)** NK Cells, **(D)** Macrophages, **(E)** B Cells, **(F)** Fibroblasts. The networks are color-coded based on the respective cluster IDs.

Supplementary Figure 3 Expression profiling of marker genes in ST-seq datasets. **(A, B)** Spatial feature plots of marker genes in two ST-seq datasets.
