# Supplementary material for: Integrative analysis reveals a four-gene signature for predicting survival and immunotherapy response in colon cancer patients using bulk and single-cell RNA-seq data
Source: Front Oncol. 2023 Oct 31;13:1277084. doi: 10.3389/fonc.2023.1277084 (PMC10644708; doi:10.3389/fonc.2023.1277084)
Supplement: Supplementary Figure 1 — Automated annotation using “singleR”. (A) Overview of Gene Count and Mitochondrial Gene Proportion in All Samples. (B) Detailed information on each scRNA-seq sample downloaded in this study. (C) UAMP plot based on “singleR” annotation. (D) Dot plot illustrating expression levels and percentages of marker genes for each of the clusters. UMAP, Uniform Manifold Approximation and Projection; scRNA-seq, single-cell RNA sequencing. [file DataSheet_1.pdf]

Figure S1

A

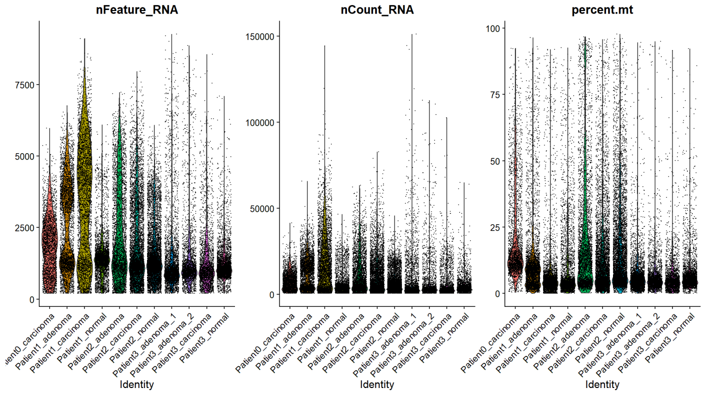

B

	Seq tech	Sample name	Patient	Tissue	Cell number	Average count
Sample_1	10X	Patient0_carcinoma	Patient0	carcinoma	1524	9422
Sample_2	10X	Patient1_adenoma	Patient1	adenoma	3082	12917
Sample_3	10X	Patient1_carcinoma	Patient1	carcinoma	2955	16639
Sample_4	10X	Patient1_normal	Patient1	normal	2960	5636
Sample_5	10X	Patient2_adenoma	Patient2	adenoma	1911	11338
Sample_6	10X	Patient2_carcinoma	Patient2	carcinoma	4589	8426
Sample_7	10X	Patient2_normal	Patient2	normal	4266	5675
Sample_8	10X	Patient3_adenoma_1	Patient3	adenoma	3605	4959
Sample_9	10X	Patient3_adenoma_2	Patient3	adenoma	2296	5289
Sample_10	10X	Patient3_carcinoma	Patient3	carcinoma	2311	5645
Sample_11	10X	Patient3_normal	Patient3	normal	3714	3755

C

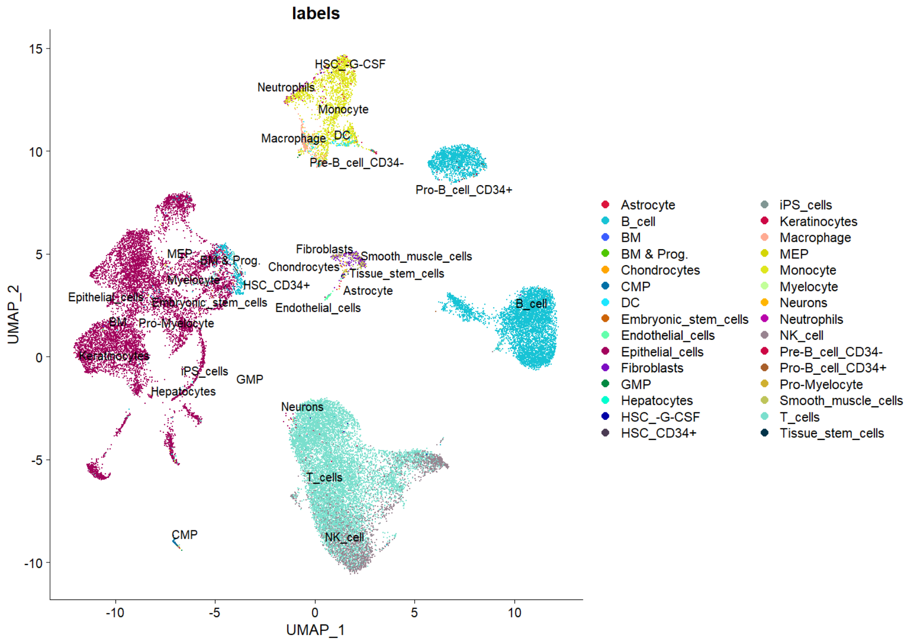

D

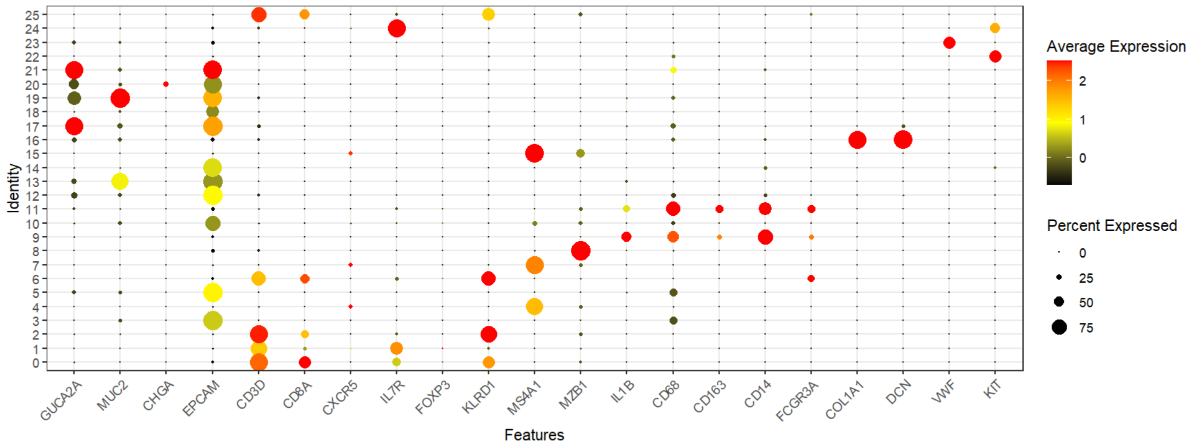

Figure S2

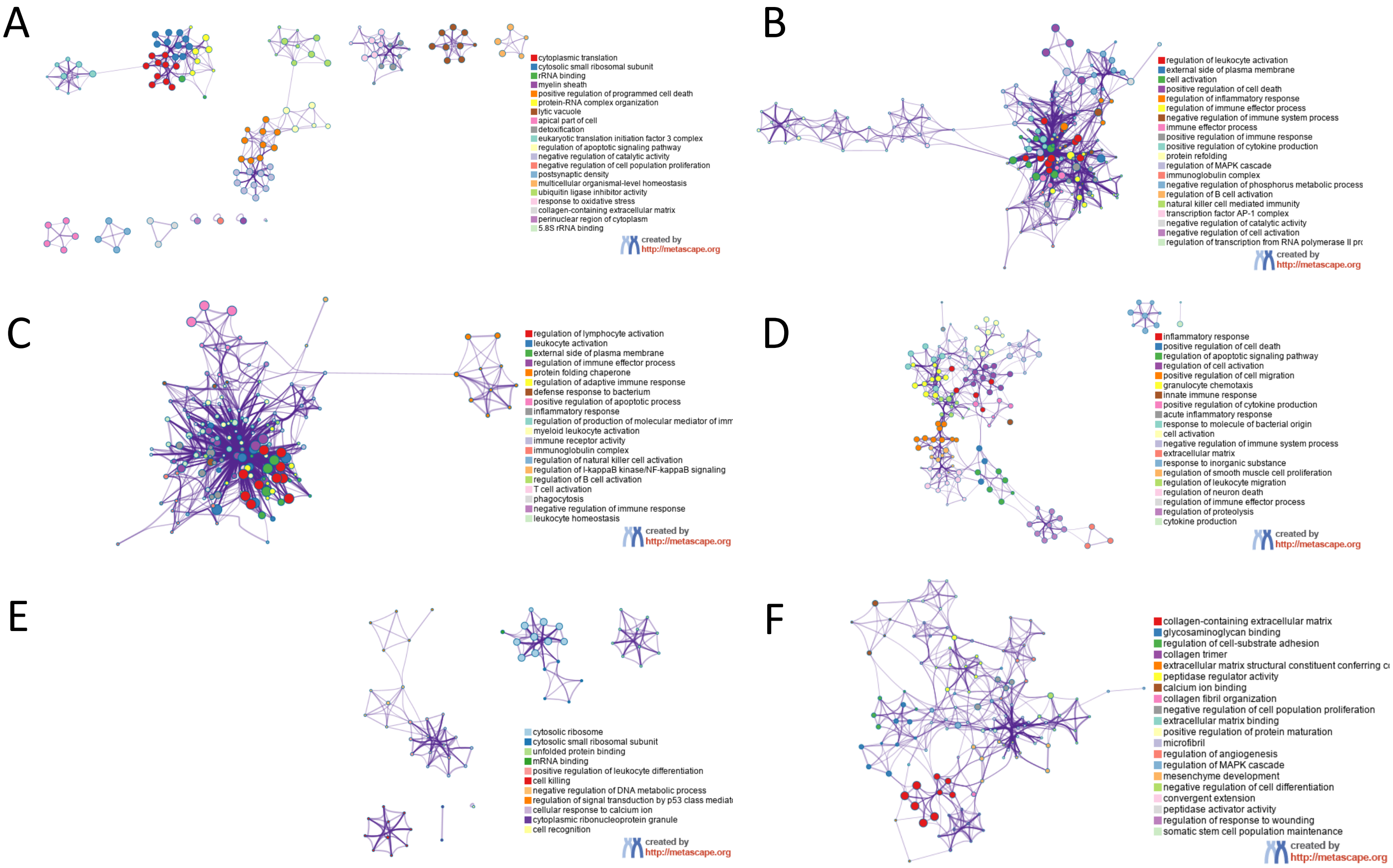
A
cytoplasmic translation
cytosolic small ribosomal subunit
rRNA binding
myelin sheath
positive regulation of programmed cell death
protein-RNA complex organization
lytic vacuole
apical part of cell
detoxification
eukaryotic translation initiation factor 3 complex
regulation of apoptotic signaling pathway
negative regulation of catalytic activity
negative regulation of cell population proliferation
postsynaptic density
multicellular organismal-level homeostasis
ubiquitin ligase inhibitor activity
response to oxidative stress
collagen-containing extracellular matrix
perinuclear region of cytoplasm
5.8S rRNA binding
created by
http://metascape.org
B
regulation of leukocyte activation
external side of plasma membrane
cell activation
positive regulation of cell death
regulation of inflammatory response
regulation of immune effector process
negative regulation of immune system process
immune effector process
positive regulation of immune response
positive regulation of cytokine production
protein refolding
regulation of MAPK cascade
immunoglobulin complex
negative regulation of phosphorus metabolic process
regulation of B cell activation
natural killer cell mediated immunity
transcription factor AP-1 complex
negative regulation of catalytic activity
negative regulation of cell activation
regulation of transcription from RNA polymerase II pro
created by
http://metascape.org
C
regulation of lymphocyte activation
leukocyte activation
external side of plasma membrane
regulation of immune effector process
protein folding chaperone
regulation of adaptive immune response
defense response to bacterium
positive regulation of apoptotic process
inflammatory response
regulation of production of molecular mediator of imm
myeloid leukocyte activation
immune receptor activity
immunoglobulin complex
regulation of natural killer cell activation
regulation of I-kappaB kinase/NF-kappaB signaling
regulation of B cell activation
T cell activation
phagocytosis
negative regulation of immune response
leukocyte homeostasis
created by
http://metascape.org
D
inflammatory response
positive regulation of cell death
regulation of apoptotic signaling pathway
regulation of cell activation
positive regulation of cell migration
granulocyte chemotaxis
innate immune response
positive regulation of cytokine production
acute inflammatory response
response to molecule of bacterial origin
cell activation
negative regulation of immune system process
extracellular matrix
response to inorganic substance
regulation of smooth muscle cell proliferation
regulation of leukocyte migration
regulation of neuron death
regulation of immune effector process
regulation of proteolysis
cytokine production
created by
http://metascape.org
E
cytosolic ribosome
cytosolic small ribosomal subunit
unfolded protein binding
mRNA binding
positive regulation of leukocyte differentiation
cell killing
negative regulation of DNA metabolic process
regulation of signal transduction by p53 class mediat
cellular response to calcium ion
cytoplasmic ribonucleoprotein granule
cell recognition
created by
http://metascape.org
F
collagen-containing extracellular matrix
glycosaminoglycan binding
regulation of cell-substrate adhesion
collagen trimer
extracellular matrix structural constituent conferring c
peptidase regulator activity
calcium ion binding
collagen fibril organization
negative regulation of cell population proliferation
extracellular matrix binding
positive regulation of protein maturation
microfibril
regulation of angiogenesis
regulation of MAPK cascade
mesenchyme development
negative regulation of cell differentiation
convergent extension
peptidase activator activity
regulation of response to wounding
somatic stem cell population maintenance
created by
http://metascape.org

Figure S3

A

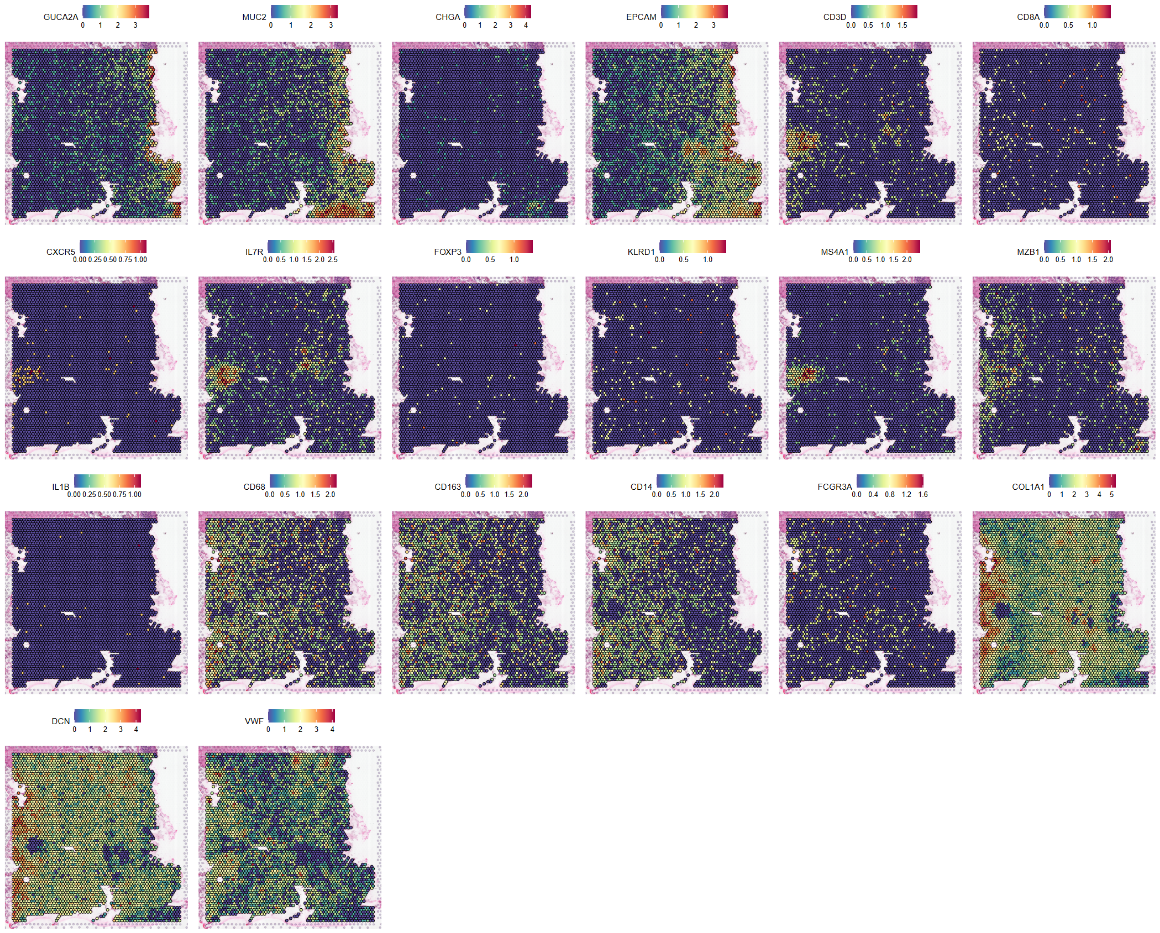

GUCA2A 0 1 2 3
MUC2 0 1 2 3
CHGA 0 1 2 3 4
EPCAM 0 1 2 3
CD3D 0.0 0.5 1.0 1.5
CD8A 0.0 0.5 1.0
CXCR5 0.00 0.25 0.50 0.75 1.00
IL7R 0.0 0.5 1.0 1.5 2.0 2.5
FOXP3 0.0 0.5 1.0
KLRD1 0.0 0.5 1.0
MS4A1 0.0 0.5 1.0 1.5 2.0
MZB1 0.0 0.5 1.0 1.5 2.0
IL1B 0.00 0.25 0.50 0.75 1.00
CD68 0.0 0.5 1.0 1.5 2.0
CD163 0.0 0.5 1.0 1.5 2.0
CD14 0.0 0.5 1.0 1.5 2.0
FCGR3A 0.0 0.4 0.8 1.2 1.6
COL1A1 0 1 2 3 4 5
DCN 0 1 2 3 4
VWF 0 1 2 3 4

B

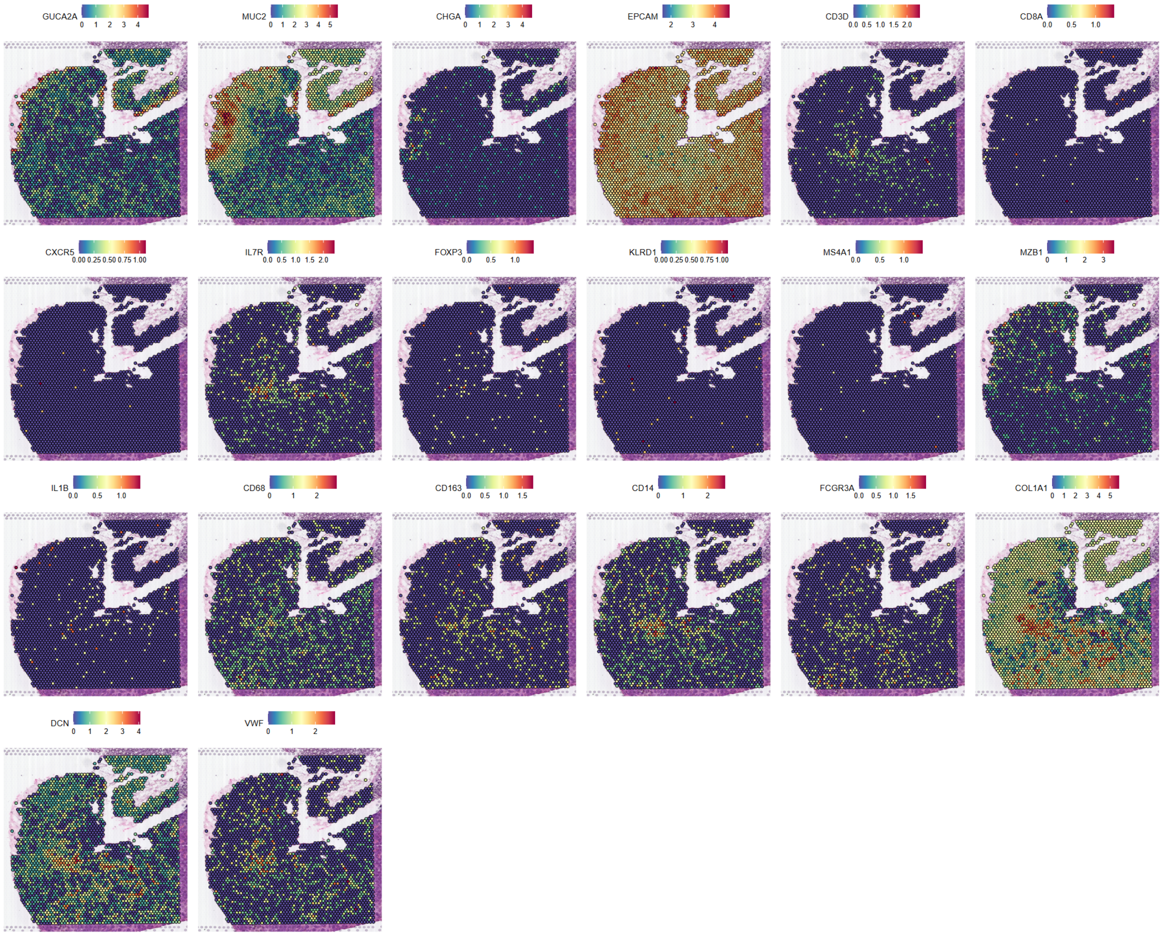

GUCA2A 0 1 2 3 4
MUC2 0 1 2 3 4 5
CHGA 0 1 2 3 4
EPCAM 2 3 4
CD3D 0.0 0.5 1.0 1.5 2.0
CD8A 0.0 0.5 1.0
CXCR5 0.00 0.25 0.50 0.75 1.00
IL7R 0.0 0.5 1.0 1.5 2.0
FOXP3 0.0 0.5 1.0
KLRD1 0.00 0.25 0.50 0.75 1.00
MS4A1 0.0 0.5 1.0
MZB1 0 1 2 3
IL1B 0.0 0.5 1.0
CD68 0 1 2
CD163 0.0 0.5 1.0 1.5
CD14 0 1 2
FCGR3A 0.0 0.5 1.0 1.5
COL1A1 0 1 2 3 4 5
DCN 0 1 2 3 4
VWF 0 1 2
